# A First Metabolite Analysis of Norfolk Island Pine Resin and Its Hepatoprotective Potential to Alleviate Methotrexate (MTX)-Induced Hepatic Injury

**DOI:** 10.3390/ph17070970

**Published:** 2024-07-22

**Authors:** Sherouk Hussein Sweilam, Dalia E. Ali, Ahmed M. Atwa, Ali M. Elgindy, Aya M. Mustafa, Manar M. Esmail, Mahmoud Abdelrahman Alkabbani, Mohamed Magdy Senna, Riham A. El-Shiekh

**Affiliations:** 1 Department of Pharmacognosy, College of Pharmacy, Prince Sattam Bin Abdulaziz University, Al-Kharj 11942, Saudi Arabia; s.sweilam@psau.edu.sa; 2 Department of Pharmacognosy, Faculty of Pharmacy, Egyptian Russian University, Cairo-Suez Road, Badr City 11829, Egypt; 3 Pharmacognosy and Natural Products Department, Faculty of Pharmacy, Pharos University, Alexandria 21648, Egypt; dalia.elsheikh@pua.edu.eg; 4 Department of Pharmacology and Toxicology, Faculty of Pharmacy, Egyptian Russian University, Badr City 11829, Egypt; ahmed-atwa@eru.edu.eg (A.M.A.); ali-elgindy@eru.edu.eg (A.M.E.); aya-mustafa@eru.edu.eg (A.M.M.); manar-esmail@eru.edu.eg (M.M.E.); mahmoud-kabbani@eru.edu.eg (M.A.A.); mohamed-magdymohamed@eru.edu.eg (M.M.S.); 5 Department of Pharmacognosy, Faculty of Pharmacy, Cairo University, Cairo 11562, Egypt

**Keywords:** *Araucaria heterophylla*, diterpenes, IL-6/JAK/STAT3 pathway, methotrexate-induced liver injury, resin, TGF-β/NF-κB signaling cascade

## Abstract

Drug-induced liver injury (DILI) represents a significant clinical challenge characterized by hepatic dysfunction following exposure to diverse medications. Methotrexate (MTX) is a cornerstone in treating various cancers and autoimmune disorders. However, the clinical utility of MTX is overshadowed by its ability to induce hepatotoxicity. The current study aims to elucidate the hepatoprotective effect of the alcoholic extract of Egyptian *Araucaria heterophylla* resin (AHR) on MTX-induced liver injury in rats. AHR (100 and 200 mg/kg) significantly decreased hepatic markers (AST, ALT, and ALP), accompanied by an elevation in the antioxidant’s markers (SOD, HO-1, and NQO1). AHR extract also significantly inhibited the TGF-β/NF-κB signaling pathway as well as the downstream cascade (IL-6, JAK, STAT-3, and cyclin D). The extract significantly reduced the expression of VEGF and p38 with an elevation in the BCL2 levels, in addition to a significant decrease in the IL-1β and TNF-α levels, with a prominent effect at a high dose (200 mg/kg). Using LC-HRMS/MS analysis, a total of 43 metabolites were tentatively identified, and diterpenes were the major class. This study presents AHR as a promising hepatoprotective agent through inhibition of the TGF-β/NF-κB and JAK/STAT3 pathways, besides its antioxidant and anti-inflammatory effects.

## 1. Introduction

Drug-induced liver injury (DILI) represents a significant clinical challenge characterized by hepatic dysfunction following exposure to diverse medications, including prescription and over-the-counter drugs, herbal supplements, and dietary supplements. DILI involves a heterogeneous spectrum of liver abnormalities, ranging from asymptomatic elevation of liver enzymes to acute liver failure, posing a substantial burden on public health and clinical practice [1]. The pathogenesis of DILI is multifaceted and multifactorial, involving a myriad of mechanisms such as direct hepatotoxicity, immune-mediated reactions, metabolic idiosyncrasies, and genetic predispositions [2].

Methotrexate (MTX), a folic acid analogue, is a cornerstone in treating various cancers and autoimmune disorders [3]. It has anti-proliferative and anti-inflammatory properties [4]. However, the clinical utility of MTX is overshadowed by its capability to induce hepatotoxicity, ranging from mild elevations in liver enzymes to severe liver injury, a serious concern, especially in long-term and high-dose regimens [5].

One of the central pathways involved in methotrexate-induced liver injury (MILI) is the transforming growth factor-beta (TGF-β)/nuclear factor-kappa B (NF-κB) signaling cascade [6]. TGF-β is a multifunctional cytokine that plays a pivotal role in inflammation and fibrogenesis [7]. The hallmark of MILI, liver fibrosis, is exacerbated by the deposition of extracellular matrix proteins resulting from the activation of hepatic stellate cells by TGF-β [8]. In addition to TGF-β, NF-κB emerges as a critical player in the pathogenesis of MILI [9]. NF-κB orchestrates the transcriptional activation of pro-inflammatory chemokines and cytokines like tumor necrosis factor-alpha (TNF-α), interleukin-1β (IL-1β), IL-2 and IL-6, driving the inflammatory cascade and extending liver damage [5,10,11]. Moreover, MTX was linked to the generation of oxidative stress within hepatocytes by disrupting the balance between the reactive oxygen species (ROS) and the defensive antioxidant system, enhancing the inflammatory response mediated through increased production of inflammatory cytokines such as IL-6, leading to oxidative stress-induced hepatocellular injury [12]. 

Emerging evidence suggests that ROS and IL-6 induce the activation of the janus kinase (JAK)/signal transducer and activation of transcription 3 (STAT-3) signaling pathway, which is widely involved in inflammation, apoptosis and fibrogenesis [5]. The activation of the JAK/STAT pathway triggers a downstream cascade, including the upregulation of cyclin D, a key regulator of cell progression and proliferation, vascular endothelial growth factor (VEGF), a key regulator for angiogenesis, and the p38 mitogen-activated protein kinase (MAPK) pathway, involved in cell apoptosis, fibrosis and inflammation [13,14,15].

Apoptosis is a central mechanism in the cellular injury process implicated in MILI, which arises from an overproduction of ROS that overwhelms the anti-oxidant defenses, leading to oxidative damage to cellular macromolecules and activation of apoptosis [16]. According to previous studies, MILI involves increased apoptosis due to it activating pro-apoptotic pathways, including Bax and caspase-3, and inhibiting anti-apoptotic factors such as BCL2 [17]. Moreover, another study elucidated the role of the activation of p38 MAPK, a stress-activated protein kinase, in stimulating the apoptotic process in MTX-induced toxicity [18]. The complicated relationship between ROS, inflammation, and apoptosis in MILI emphasizes the necessity for adjunctive therapies capable of mitigating these detrimental effects of MTX and improving patient outcomes in MTX therapy. 

Milk thistle has a complex mixture of flavonolignans called silymarin (SIL), which is known for its hepatoprotective impact [19]. For ages, SIL has been used in traditional medicine to treat a variety of liver disorders. Extensive research in recent decades has produced solid data confirming silymarin’s hepatoprotective benefits, with several studies proving its efficiency in preventing liver impairment caused by a wide range of insults such as toxins, medications, alcohol, and viral infections [19,20]. The hepatoprotective actions of SIL are mediated by several pathways, including antioxidant, anti-inflammatory, and anti-fibrotic properties [21].

Since the beginning of creation, humans have relied on the natural source for their food and treatment of any disorders, and the most useful source is plant species. Whole medicinal plants or their parts have been used to prevent or treat many human diseases from ancient times until nowadays, especially in developing countries, where they are most popular; around 3.3 billion people use herbal medicines as the first line of therapeutic defense [22]. 

Most *Araucaria* species that belong to family Araucariaceae are evergreen decorative plants and grow widely in China and many countries of te central and southern Americas [22]. *Araucaria heterophylla* Salisb. Franco (syn. *A. excelsa*) (Norfolk Island pine or Christmas tree plant) is exhibited to have anticancer, antidepressant, anti-inflammatory, antimicrobial, antioxidants, and antiviral effects, and traditionally, it is used as a remedy for toothache [22,23,24,25]. The Egyptian ecospecies *Araucaria heterophylla* resin (AHR) was reported to have four diterpenes (labda-8(17),14-diene, 13-epi-cupressic acid, 13-*O*-acetyl-13-epi-cupressic acid, and phyllocladanol), in addition to volatile oils, which are dominated by monoterpene hydrocarbons, and α-pinene is the most abundant compound. Other monoterpenes such as sabinene, camphene, and D-limonene are also present, albeit in lower concentrations. Minor components identified in the AHR included flavonoids and phenolic compounds [23]. The leaves of the different cultivars of this species are present in different countries such as Australia, Germany, India, Indonesia, and Hawaii, and were found to be rich with volatile oils using GC-MS analysis [26].

However, as far as our knowledge extends, there has been no full investigation of the AHR secondary metabolites of the Egyptian cultivar. Herein, our objectives were to achieve the (i) characterization of the phytochemical profile from the AHR Egyptian cultivar, (ii) assessment of the possible hepatoprotective effects of AHR against MILI in rats, and clarification of the potential mechanisms involved in these effects with a focus on the TGF-β/NF-κB/TNF-α/IL-1β pathway and IL-6/JAK/STAT-3/cyclin D/VEGF/p38 MAPK/BCL2 signaling cascade.

## 2. Results

### 2.1. Phytochemical Analysis of AHR Extract

**Figure 1:**
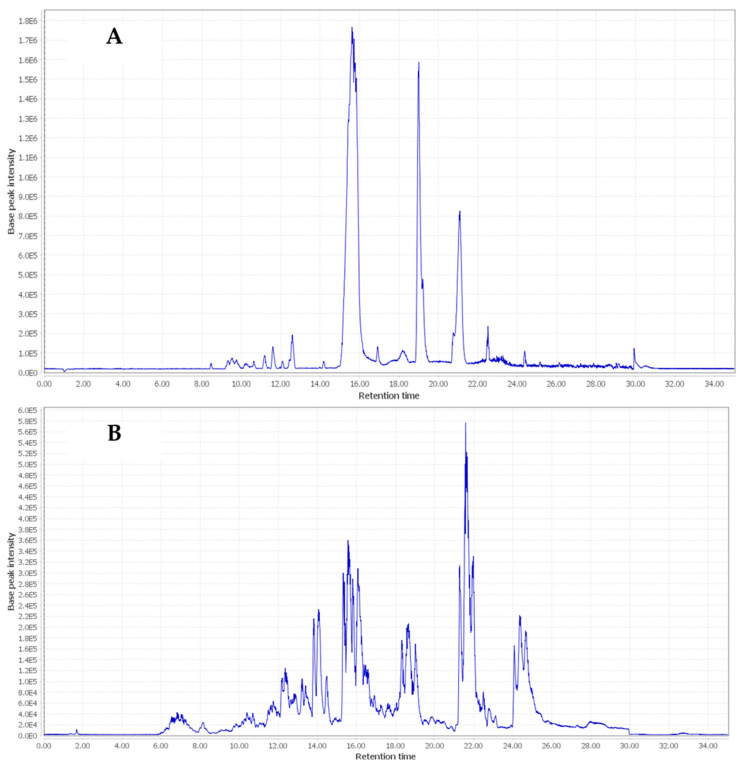
Base peak chromatograms of negative (**A**) and positive (**B**) ionization modes of total methanolic extract of *Araucaria heterophylla* resin (AHR).

**Figure 2:**
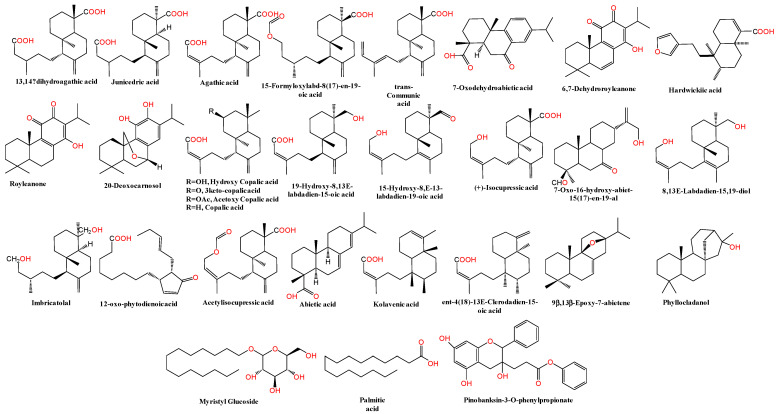
Representation of the major bioactive compounds observed in the *Araucaria heterophylla* resin (AHR).

There have previously been no previous full reports about the secondary metabolite classes or their composition in AHR. This study suggests that the qualitative profile gives new insights into the phytochemical composition of the crude alcoholic extract of the Egyptian cultivar ecospecies, AHR. The LC-HR-MS/MS device was operated in both modes to provide broad scanning coverage as reported previously [27,28,29]. From the comparison of the two ionization modes, the negative-ion spectral chromatogram showed better sensitivity and observed peaks. The base peak chromatogram (BPC) of the representative extract sample is depicted in Figure 1. Identified metabolites, retention times, and experimental and literature molecular ions and fragment ions for peaks are presented in Table 1, and their structures are illustrated in Figure 2. 

Diterpenes represent the majority of the subclasses in the AHR extract, where about 40 compounds are identified, of which 15 are tentatively reported for the first time in our plant along with two saturated and glycosidic fatty acids and one substituted flavanol. Also, among the diterpene derivatives, many metabolites in peaks 1–5, 10, 11/12, 17–25, 29, 30–32, 38/39, 41, and 43 are herein reported in the Araucaria genus for the first time. Diterpenes showed fragment ions relating to loss of water [18 Da], carbon dioxide [44 Da], C_2_H_4_O_2_ [60 Da], and/or the acetoxy group [60 Da]. Compounds **#**17–25 and 30–32 were found to be the major constituents in the total ion chromatograms in both negative and positive modes, as shown in Figure 1 and Figure 2. More evidence for compound annotations was acquired through diagnostic MS/MS fragments, ionization behavior, parent compound information and a review of the reported chemical constituents. For more thorough identification, details of the exact structures of unreported peak metabolites should be sought using other spectral tools. By comparing the RT values, UV-vis spectra, and MS patterns in both modes of the compounds published in the literature, 43 metabolites were assigned; in addition, the similarity of the fragmentation pathway was found to prevail among the recognized compounds through the chromatogram. In 2020, two of six varieties of AH were mentioned with four diterpenes, labda-8(17),14-diene, 13-epi-cupressic acid, 13-O-acetyl-13-epi-cupressic acid and phyllocladanol, from the two ecospecies Egyptian resin stem extract and Indian oil resin [25,30]. 

**Table 1:** Identified metabolites of *Araucaria heterophylla* resin (AHR) using LC- MS in negative and positive modes.

No.	Identification	R_t_ (min)	[M] Exact Mass	Experimental Ion (*m*/*z*)	Formula	Ion	Ms/Ms	Ref.
1.	13,14-dihydroagathic acid/ Labd-8(17)-en-15,19-dioic acid (Junicedric acid)	9.55	336.2300	335.2226	C_20_H_32_O_4_	[M−H]^−^	317.2110, 299.2001	[31,32]
2.		10.63	336.2300	335.2228		[M−H]^−^	317.2110, 299.2001	
3.		12.54	336.2300	335.2205		[M−H]^−^	317.2110, 299.2001	
4.		15.57	336.2300	335.2202		[M−H]^−^	317.2110, 299.2001	
5.		16.37	336.2300	335.2207		[M−H]^−^	317.2110, 299.2001	
6.	Agathic acid	10.17	334.2144	333.2060	C_20_H_30_O_4_	[M−H]^−^	315.1919, 289.1719, 273.1453	[31]
7.	15-Formyloxylabd-8(17)-en-19-oic acid	11.55	350.2457	349.2398	C_21_H_34_O_4_	[M−H]^−^	315.1971, 301.1817	[31,32]
8.		15.32	350.2457	373.2335		[M+Na]^+^	323.1999, 301.2150	
9.	trans-Communic acid	11.64	302.2245	301.2151	C_20_H_30_O_2_	[M−H]^−^	151.1190	[31,33]
10.	Dihydro-15-Acetoxy-8,9-epoxylabdane-19-oic acid/ Dihydro-15-Acetoxy-8,17-epoxylabdane-19-oic acid	11.97	378.2406	377.2334	C_22_H_34_O_5_	[M−H]^−^	316.9489, 301.1809	[32,34]
11.	7-Oxodehydroabietic acid/6,7-Dehydroroyleanone	11.88	314.1881	313.1749	C_20_H_26_O_3_	[M−H]^−^	159.0800	[35]
12.		11.57	314.1882	315.1955		[M+H]^+^	297.1862, 269.1898	
13.	Hardwickiic acid/Royleanone /20-Deoxocarnosol	11.57	316.2038	315.1955	C_20_H_28_O_3_	[M−H]^−^	297.1880, 271.2076	[36]
14.		12.56	316.2038	315.1957		[M−H]^−^	297.1880, 271.2076	
15.		13.59	316.2038	315.19746		[M−H]^−^	297.1880, 271.2076	
16.		12.36	316.2038	317.2111		[M+H]^+^	299.1913, 271.2040	
17.	Hydroxy Copalic acid/ 19-Hydroxy-8,13E-labdadien-15-oic acid/ 15-Hydroxy-8,E-13-labdadien-19-oic acid/ (+)-Isocupressic acid	11.61	320.2351	319.2256	C_20_H_32_O_3_	[M−H]^−^	301.2172, 219.1386, 217.1199	[37]
18.		12.65	320.2351	319.2253		[M−H]^−^	301.2172, 219.1386, 217.1199	
19.		13.91	320.2351	319.2253		[M−H]^−^	301.2172, 219.1386, 217.1199	
20.		15.59	320.2351	319.2257		[M−H]^−^	301.2172, 219.1386, 217.1199	
21.	3keto-copalic acid/ 12-Oxolabda-8(17),13E-dien-19 oic acid/ 7-Oxo-16-hydroxy-abiet-15(17)-en-19-al	11.92	318.2194	317.2095	C_20_H_30_O_3_	[M−H]^−^	299.2042, 273.1555	[38]
22.		12.36	318.2194	317.2111		[M−H]^−^	299.2042, 273.1555	
23.		13.56	318.2194	317.2098		[M−H]^−^	299.2042, 273.1555	
24.		15.81	318.2194	317.2105		[M−H]^−^	299.2042, 273.1555	
25.		12.65	318.2194	319.2253		[M+H]^+^	301.2151, 273.2099	
26.	8,13E-Labdadien-15,19-diol/Imbricatolal	11.95	306.2558	305.2461	C_20_H_34_O_2_	[M−H]^−^	287.2350, 257.2278	[32]
27.		17.17	306.2558	305.2461		[M−H]^−^	287.2350, 257.2278	
28.		16.86	306.2558	305.2461		[M−H]^−^	287.2350, 257.2278	
29.	12-oxo-phytodienoic acid	14.19	292.2038	291.1967	C_18_H_28_O_3_	[M−H]^−^	249.0	[39]
30.	Acetoxy Copalic acid/Acetylisocupressic acid	19.01	362.2457	361.2386	C_22_H_34_O_4_	[M−H]^−^	301.2167, 219.1764, 217.8029, 189.6338	[37]
31.		20.51	362.2457	361.2388		[M−H]^−^	301.2167, 219.1764, 217.8029, 189.6338	
32.		20.80	362.2457	361.2385		[M−H]^−^	301.2167, 219.1764, 217.8029, 189.6338	
33.	Abietic acid	21.08	302.2245	301.2175	C_20_H_30_O_2_	[M−H]^−^	283.2650, 265.1478, 227.1994	[40]
34.		22.45	302.2245	301.2174		[M−H]^−^	283.2650, 265.1478, 227.1994	
35.		22.96	302.2245	301.2176		[M−H]^−^	283.2650, 265.1478, 227.1994	
36.		21.29	302.2245	303.2306		[M+H]^+^	285.2190, 267.2028, 227.1756	
37.	Copalic acid/Kolavenic acid/ent-4(18)-13E-Clerodadien-15-oic acid	21.59	304.2402	303.2306	C_20_H_32_O_2_	[M−H]^−^	259.2420, 219.1381	[40]
38.	9β,13β-Epoxy-7-abietene	22.76	288.2453	287.2519	C_20_H_32_O	[M−H]^−^	215.1700	[41]
39.		22.76	288.2453	289.2517		[M+H]^+^	215.1700	
40.	Phyllocladanol	23.12	290.2609	289.2152	C_20_H_34_O	[M−H]^−^	149.1295	[42]
41.	Myristyl Glucoside	22.51	376.2824	375.2753	C_20_H_40_O_6_	[M−H]^−^	301.2170, 255.2336	[43]
42.	Palmitic acid	22.82	256.2402	255.2330	C_16_H_32_O_2_	[M−H]^−^	255.2334	[31]
43.	Pinobanksin-3-*O*-phenylpropionate	24.49	404.3137	403.3046	C_24_H_20_O_6_	[M−H]^−^	271.2021, 253.0872	[44]

### 2.2. Effect of AHR on Hepatic Markers

**Figure 3:**
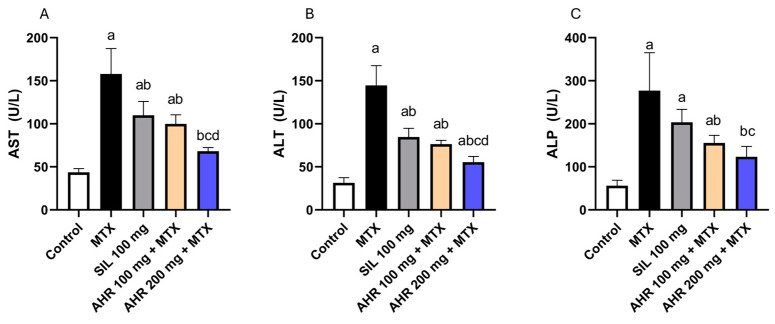
Influence of AHR on hepatic injury biomarkers. (**A**) AST, (**B**) ALT, and (**C**) ALP. Results are displayed as mean +/− SD (number per group = 6 rats). a: significant vs. normal control group, b: significant vs. MTX group, c: significant vs. (SIL 100 mg + MTX) group, d: significant vs. (AHR 100 mg + MTX) group. Statistical analysis was conducted using ANOVA followed by Tukey’s post hoc test at a *p* value < 0.05. MTX, methotrexate; SIL, silymarin; AHR, *Araucaria heterophylla* resin; ALT, alanine transaminase; AST, aspartate transaminase; ALP, alkaline phosphate.

Methotrexate exhibited a significant increase in the levels of AST, ALT, and ALP by 3.62, 4.6, and 4.93 times, respectively, compared to the control group. In contrast, administration of AHR (100 mg) resulted in a significant reduction in serum levels of AST, ALT, and ALP by 36.72%, 47.14%, and 43.87%, respectively, compared to those in the MTX group. In addition, treatment with AHR (200 mg) resulted in significant reductions of 56.76%, 61.67%, and 55.42% in the levels of AST, ALT, and ALP, respectively, compared to those in the MTX group. In addition, administration of AHR at a dosage (200 mg) resulted in a significant decrease in the serum level of AST and ALT by 31.67% and 27.5%, respectively, when compared to those in the AHR (100 mg) group. Notably, the group treated with Silymarin (100 mg) showed the same pattern compared to the MTX group, with significant decreases of 30.36% and 41.32% in the serum levels of AST and ALT, respectively (Figure 3).

### 2.3. Effect of AHR on Antioxidant Parameters

**Figure 4:**
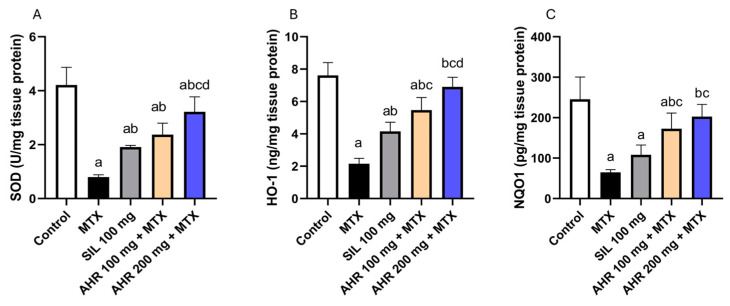
Influence of AHR on antioxidant markers. (**A**) SOD, (**B**) HO-1, and (**C**) NQO1. Results are displayed as the mean +/− SD (number per group = 6 rats). a: significant vs. control group, b: significant vs. MTX group, c: significant vs. (SIL 100 mg + MTX) group, d: significant vs. (AHR 100 mg + MTX) group. Statistical analysis was conducted using ANOVA followed by Tukey’s post hoc test at a *p* value < 0.05. MTX, methotrexate; SIL, silymarin; AHR, *Araucaria heterophylla* resin; SOD, superoxide dismutases; HO-1, heme oxygenase-1; NQO1, NAD(P)H dehydrogenase [quinone] 1.

Methotrexate induced a significant reduction in the levels of SOD, HO-1, and NQO1 by 81.03%, 71.66%, and 73.55%, respectively, compared to those of the normal group. whereas AHR (100 mg) treatment displayed a significant elevation in SOD, HO-1, and NQO1 levels by 2.96, 2.53, and 2.65 times, respectively, compared to those in the MTX group. In addition, the administration of AHR (200 mg) resulted in a significant increase in the levels of SOD, HO-1, and NQO1 by 4.03, 3.2, and 3.11 times, respectively, compared to those in the MTX group. Furthermore, the administration of AHR (200 mg) led to a significant 1.35-fold increase in SOD and a 1.26-fold increase in HO-1, compared to the AHR (100 mg) group. Similarly, administration of Silymarin (100 mg) resulted in an equivalent pattern, with a significant rise in SOD and HO-1 levels by 2.39 and 1.92 times, respectively, compared to those in the MTX group (Figure 4). Potent antioxidant activity was observed, suggesting the extract’s ability to neutralize harmful free radicals and protect against oxidative damage.

### 2.4. Effect of AHR on NF-κB, IL-1β, IL-6 and TNF-α

**Figure 5:**
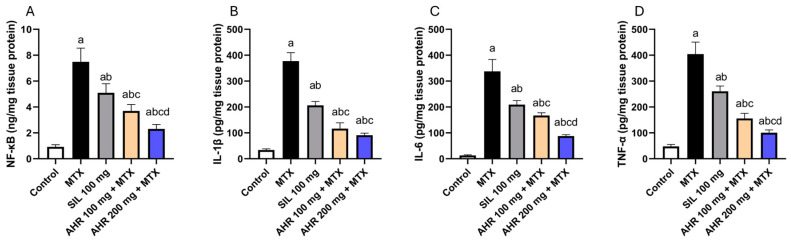
Influence of AHR on tissue content of NF-κB, IL-1β, IL-6 and TNF-α. (**A**) NF-κB, (**B**) IL-1β, (**C**) IL-6, (**D**) TNF-α. Data are displayed as the mean +/− SD (number per group = 6 rats). a: significant vs. control group, b: significant vs. MTX group, c: significant vs. (SIL 100 mg + MTX) group, d: significant vs. (AHR 100 mg + MTX) group. Statistical analysis was conducted using ANOVA followed by Tukey’s post hoc test at a *p* value < 0.05. MTX, methotrexate; SIL, silymarin; AHR, *Araucaria heterophylla* resin; NF-κB, nuclear factor-kappa B; IL-1β, interleukin-1β; IL-6, interleukin-6; TNF-α, tumor necrosis factor alpha.

Methotrexate injection exhibited a significant elevation in NF-κB, IL-1β, IL-6, and TNF-α levels, by 8.13, 11.07, 26.15, and 8.43 times, respectively, compared to the control group. Nevertheless, the administration of AHR (100 mg) resulted in a significant decrease in the liver content of NF-κB, IL-1β, IL-6, and TNF-α by 50.56%, 69.12%, 50.4%, and 61.55%, respectively, as compared to the MTX group. In addition, the administration of AHR (200 mg) resulted in a significant reduction in the levels of NF-κB (59.21%), IL-1β (75.89%), IL-6 (74.02%), and TNF-α (75.12%), compared to the group treated with MTX. Similarly, the administration of AHR (200 mg) led to a significant decrease in the levels of NF-κB (37.7%), IL-6 (47.61%), and TNF-α (35.28%) compared to the group treated with AHR (100 mg). Similarly, administering silymarin (100 mg) contributed to a significant drop in the levels of NF-κB, IL-1β, IL-6, and TNF-α by 31.93%, 37.94%, 45.3%, and 35.54%, respectively, compared to the MTX group (Figure 5). The findings overall suggest that this extract could be a valuable natural source for developing innovative treatments targeting oxidative stress and inflammatory conditions.

### 2.5. Effect of AHR on JAK, STAT3 and Cyclin D Signaling

**Figure 6:**
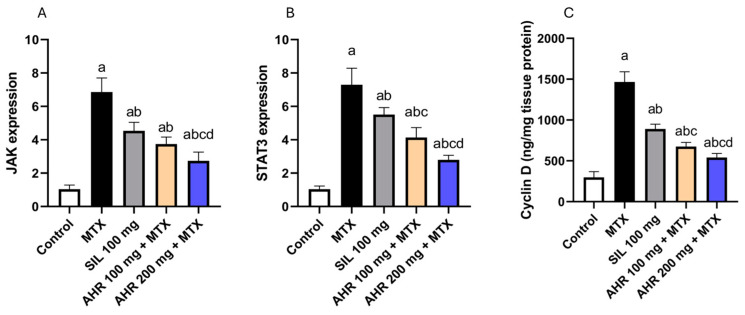
Influence of AHR on JAK, STAT3 and Cyclin D expression. (**A**) JAK, (**B**) STAT3, and (**C**) Cyclin D. Data are displayed as the mean +/− SD (number per group = 6 rats). a: significant vs. control group, b: significant vs. MTX group, c: significant vs. (SIL 100 mg + MTX) group, d: significant vs. (AHR 100 mg + MTX) group. Statistical analysis was conducted using ANOVA followed by Tukey’s post hoc test at a *p* value < 0.05. MTX, methotrexate; SIL, silymarin; AHR, *Araucaria heterophylla* resin; JAK, Janus kinase; STAT3, signal transducer and activator of transcription 3.

The administration of MTX triggered a significant rise in the expression and levels of JAK, STAT3, and Cyclin D by 6.6, 7.05, and 4.93 times, respectively, in comparison to the control group. However, treatment with AHR (100 mg) exhibited a significant decline in the expression and levels of JAK, STAT3, and Cyclin D by 45.44%, 43.28%, and 53.87%, respectively, compared to those in the MTX group. Correspondingly, treatment with AHR (200 mg) resulted in a significant decrease in the expression and content of JAK, STAT3, and Cyclin D by 60.14%, 61.7%, and 63.07%, respectively, compared to those in the MTX group. In addition, the administration of AHR (200 mg) resulted in a significant decrease in the protein expression and content of JAK, STAT3, and Cyclin D to 26.95%, 32.47%, and 19.93%, respectively, compared to AHR (100 mg). Remarkably, the administration of silymarin (100 mg) contributed to a significant drop in the expression and content of JAK, STAT3, and Cyclin D by 33.86%, 24.35%, and 39.2%, respectively, compared to those in the MTX group (Figure 6).

### 2.6. Effect of AHR on p38 and BCL2

**Figure 7:**
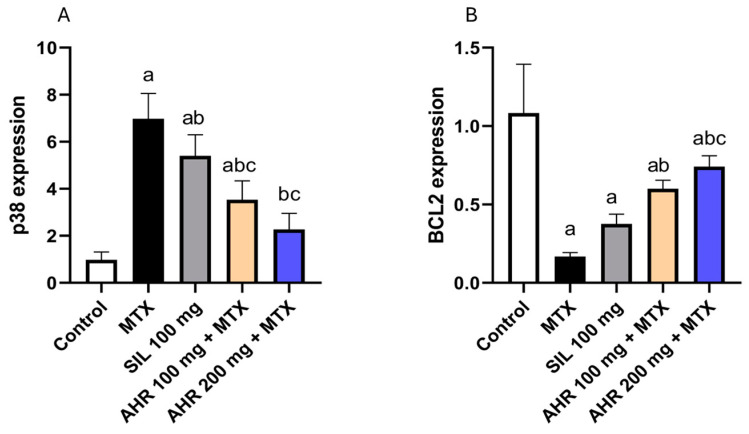
Effect of AHR on p38 and BCL2 expression. (**A**) p38, (**B**) BCL2. Data are displayed as the mean +/− SD (number per group = 6 rats). a: signficane vs. control group, b: significant vs. MTX group, c: significant vs. (SIL 100 mg + MTX) group. Statistical analysis was conducted using ANOVA followed by Tukey’s post hoc test at a *p* value < 0.05. MTX, methotrexate; SIL, silymarin; AHR, *Araucaria heterophylla* resin; BCL2, B-cell lymphoma 2.

The administration of MTX provoked a significant, 7.13-fold, rise in the expression of p38, along with a significant reduction in the expression of BCL2 by 84.69%, as compared with those in the control group. However, the administration of AHR (100 mg) caused a significant decrease in p38 expression by 49.28%, coupled with a significant, 3.62-fold, rise in BCL2 expression, compared to those in the MTX group. Similarly, the treatment with AHR (200 mg) caused a significant decrease in p38 expression by 67.44%, as well as a significant, 4.47-fold, increase in BCL2 expression, compared to those in the MTX group. Interestingly, the administration of silymarin (100 mg) contributed to a significant drop of 22.6% in p38 expression, compared to that in the group treated with MTX (Figure 7).

### 2.7. Effect of AHR on MTX-Induced Histopathological Alterations

**Figure 8:**
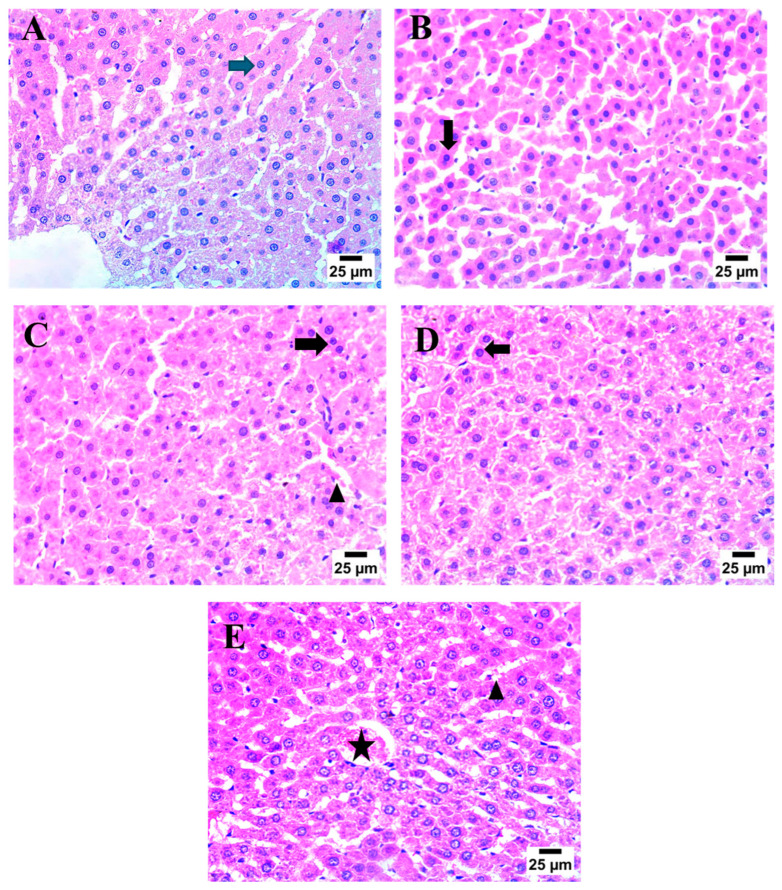
Effect of AHR on MTX-induced histopathological alterations. (**A**–**E**) Photomicrographs representing staining of hepatocytes with H & E (Scale bar 25 μm). (**A**) Control group, (**B**) MTX group, (**C**) SIL 100 mg + MTX-treated group, (**D**) AHR (100 mg)-treated group, and (**E**) AHR (200 mg)-treated group. Normal histological structure of hepatocytes (blue arrow), nuclear pyknosis in hepatocytes (black arrow), hepatic sinusoids engorged with blood (arrowhead), and congestion of the central vein (star). MTX, methotrexate; SIL, silymarin; AHR, *Araucaria heterophylla* resin.

To determine the protective impact of AHR on histopathological changes, hematoxylin and eosin (H & E) staining was utilized. Control samples demonstrated the normal histological structure of the central vein and hepatocytes (blue arrow). On the contrary, the MTX group showed severe nuclear pyknosis in hepatocytes (black arrow). On the other hand, treatment with AHR (100 mg) displayed moderate nuclear pyknosis in some hepatocytes (black arrow). At the same time, treatment with AHR (200 mg) showed congestion of the central vein (star) and mild blood engorgement in hepatic sinusoids (arrowhead). Meanwhile, treatment with silymarin (100 mg) demonstrated an increase in some hepatic sinusoids, which were engorged with blood (arrowhead), as well as in the presence of severe nuclear pyknosis in hepatocytes (black arrow) (Figure 8).

### 2.8. Effect of AHR on MTX-Induced Changes in TGF-β and VEGF Immunoreactivity

**Figure 9:**
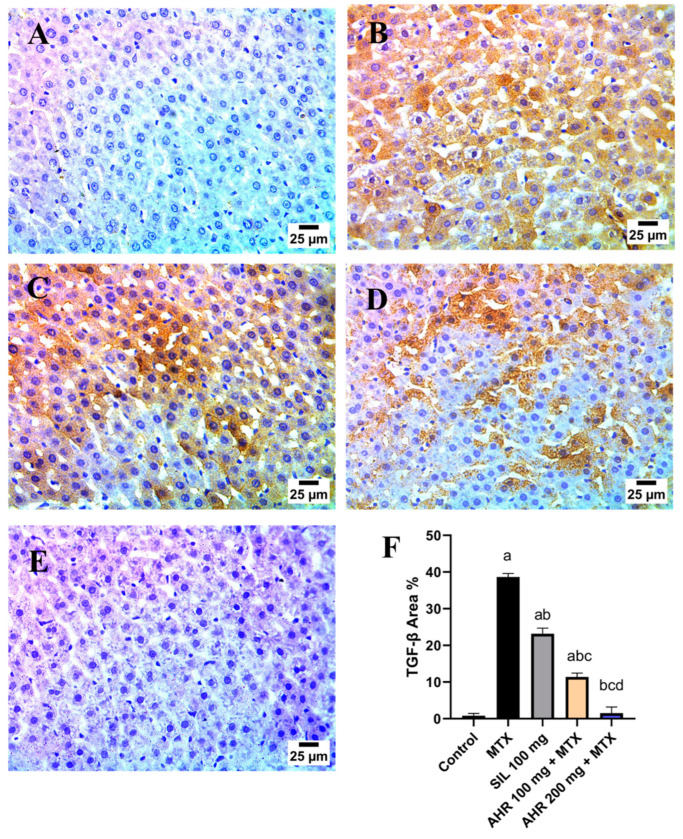
Effect of AHR on MTX-induced changes in TGF-β immunoreactivity. (**A**–**F**) Photomicrographs representing immunohistochemical analysis of TGF-β (Scale bar 25 μm). (**A**) Control group, (**B**) MTX group, (**C**) SIL 100 mg + MTX-treated group, (**D**) AHR (100 mg)-treated group, (**E**) AHR (200 mg)-treated group, and (**F**) % area of TGF-β immunoexpression. Data are displayed as the mean ± SD (number per group = 6 rats) using one-way ANOVA followed by Tukey’s post hoc test; *p* value < 0.05. a vs. control group, b vs. MTX group, c vs. (SIL 100 mg + MTX) group, and d vs. (AHR 100 mg + MTX) group. MTX, methotrexate; SIL, silymarin; AHR, *Araucaria heterophylla* resin; TGF-β, transforming growth factor-beta.

**Figure 10:**
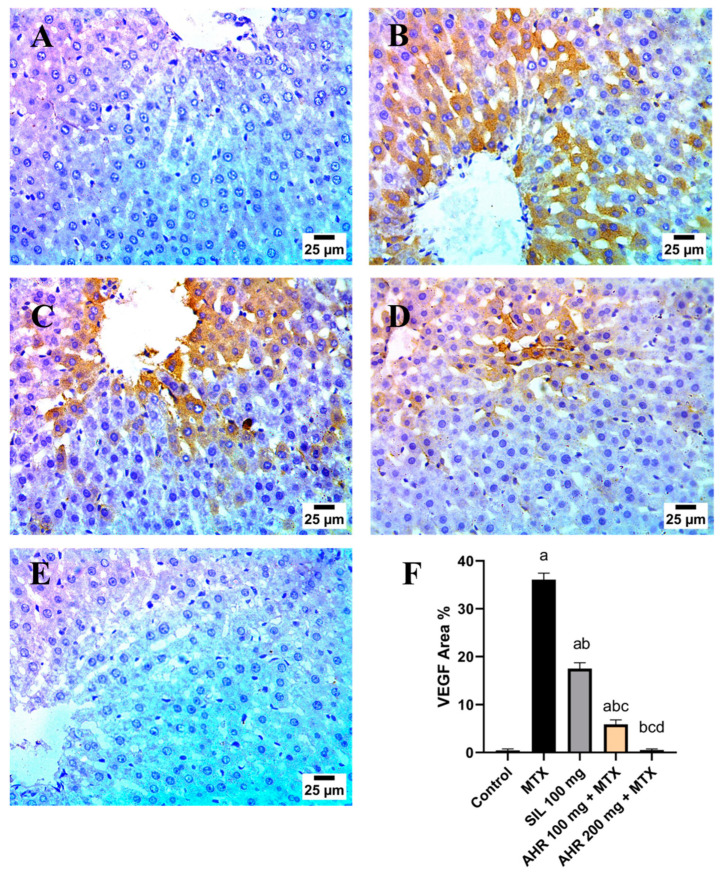
Effect of AHR on MTX-induced changes in VEGF immunoreactivity. (**A**–**F**) Photomicrographs representing immunohistochemical analysis of VEGF (scale bar: 25 μm). (**A**) Control group, (**B**) MTX group, (**C**) SIL 100 mg + MTX-treated group, (**D**) AHR (100 mg)-treated group, (**E**) AHR (200 mg)-treated group, and (**F**) % area of VEGF immunoexpression. Data are displayed as the mean ± SD (number per group = 6 rats) using one-way ANOVA followed by Tukey’s post hoc test; *p* value < 0.05. a vs. control group, b vs. MTX group, c vs. (SIL 100 mg + MTX) group, and d vs. (AHR 100 mg + MTX) group. MTX, methotrexate; SIL, silymarin; AHR, *Araucaria heterophylla* resin; VEGF, vascular endothelial growth factor.

MTX induced changes in the immunoreactivity of TGF-β (Figure 9) and VEGF (Figure 10). Control sections exhibited a downregulation in TGF-β and VEGF, while MTX injection caused a marked increase in both parameters’ immunoreactivity by 99.86 and 77.42 times, respectively, in comparison to normal group. Conversely, administration of AHR (100 mg) and (200 mg) ameliorated this elevation and decreased TGF-β and VEGF immunoreactivity to 70.53%, 83.66%, 98.85%, and 98.61%, respectively, in comparison with the MTX group. Moreover, the AHR (200 mg) group displayed a marked inhibition of TGF-β and VEGF to 96.1% and 91.5% as compared to AHR (100 mg). Meanwhile, the silymarin (100 mg)-treated group showed significant reductions in TGF-β and VEGF to 40.01% and 51.39% in comparison to those in the MTX group.

## 3. Discussion

The current study highlights the first evidence for the hepatoprotective impact of AHR on MTX-induced liver injury in rats, which was supported by diverse actions that manifested in the alleviation of serum liver function markers, and for its positive impact on histopathological changes. Its hepatoprotective effect was mediated through the inhibition of the TGF-β/NF-κB/TNF-α/IL-1β cascade and IL-6/JAK/STAT3 signaling pathway with its downstream cyclin D1/VEGF/p38 MAPK axis. In addition to this, increased activity of the antioxidant enzymes SOD, HO-1, and NQO1, as well as increased gene expression of the anti-apoptotic BCL2 protein, was observed.

In the first part of the chromatogram (Figure 1), the Labdanes-type diterpenes or their epoxy basic structures [45] appeared at RT = 9.55–11.97, 12.54, 15.32, 15.57, and 16.37 min, as revealed from the MS spectral data. Peaks 1/5, 6, 7/8, 9 and 10; [M−H]^−^ *m*/*z* at 335.2202-335.2228, 333.2060, 349.2398, 301.2151, and 377.2334 were readily annotated to identify 8 labdanes as 13,14-dihydroagathic acid/junicedric acid [29], agathic acid [29], 15-formyloxylabd-8(17)-en-19-oic acid [31], trans-communic acid [29], and dihydro-15-Acetoxy-8,17-epoxylabdane-19-oic acid/dihydro-15-Acetoxy-8,17-epoxylabdane-19-oic acid, respectively. Briefly, both junicedric acid and 15-formyloxylabd-8(17)-en-19-oic acid are considered gastroprotective and cytotoxic metabolites [32], while communic acid was found to have anti-inflammatory, anti-mycobacterial, antioxidant, cytotoxic, hypolipidemic activities, with testosterone 5α-reductase-inhibitory and relaxant effects [30,46]. Similarly, agathic acid is exhibited as an antihepatotoxic metabolite [47]. Dehydrated derivatives of dihydro-15-Acetoxy-8,9-epoxylabdane-19-oic acid and dihydro-15-Acetoxy-8,17-epoxylabdane-19-oic acid showed strong gastroprotective and cytotoxicity effects [32]. Two new identified phenolic diterpenes, were found in *Araucaria*: 7-oxodehydroabietic acid and 6,7-dehydroroyleanone. On the other hand, 6,7-dehydroroyleanone was observed previously as a cytotoxic and gastroprotective agent [48]. Two compounds were first identified in *Araucaria* genus, including 7-oxo-16-hydroxy-abiet-15(17)-en-19-al at [M−H]^−^ *m*/*z* 317.2095–319.2253 (C_20_H_30_O_3_), in peaks 21-25 (RT = 11.92–15.81 min), which is known as a potential cytotoxic agent against *A. salina* larvae [38]. Also, volatile oil 9β,13β-epoxy-7-abietene was observed at RT = 22.76 min; [M−H]^−^ *m*/*z* at 287.2519–289.2517 (C_20_H_32_O) in peaks 38,39 showed high cytotoxic potential on HCT-8, MDA-MB-435, and SF-295 cell lines [49]. One of the identified metabolite, royleanone (phenolic diterpene), was shown in peaks 13–16 (RT = 11.57–13.59 min) at [M−H]^−^ *m*/*z* 315.19746–317.2111 (C_20_H_28_O_3_) as an abietane type, and it has been determined before to have anticancer activity in *Araucaria* species[45]. On the other hand, abietane-type abietic acid was obtained from *Araucaria* genus [30], and it orally ameliorated psoriasis-like inflammation and modulated gut microbiota in in vivo assays [50]. 

Concisely, eight diterpenes, (+)-isocupressic acid, copalic acid, hydroxycopalic acid, acetylisocupressic acid, kolavenic acid, ent-4(18)-13E-clerodadien-15-oic acid, imbricatolal, and 8,13E-labdadien-15,19-diol, are also commonly found in *Araucaria* genus; they are distributed all over the chromatogram chart [30,32,51,52,53]. Recently, copalic acid was investigated and determined to have potential anti-inflammatory, antimicrobial, antiparasitic, and cytotoxic pharmacological effects [51]. On the other hand, hydroxycopalic acid was reported to exhibit antileishmanial activity [54], and finally 8,13E-labdadien-15,19-diol was identified as an antifungal component [52]. 

Furthermore, only two clerodane-type diterpenes were detected in peak 37 at RT = 21.59 min at [M-H]^−^ *m*/*z* at 303.2306 and Mol. Wt. = C_20_H_32_O_2_, including kolavenic acid [30] and ent-4(18)-13E-clerodadien-15-oic acid, which has been shown to be present in *Araucaria bidwillii* [53].

Consequently, it is worth noting that nine diterpene compounds were detected as the following aforementioned majors: hydroxycopalic acid, acetylisocupressic acid, acetoxy copalic acid; 3keto-copalic acid, (+)-isocupressic acid, 19-hydroxy-8,13E-labdadien-15-oic acid, 15-hydroxy-8,E-13-labdadien-19-oic acid, 7-oxo-16-hydroxy-abiet-15(17)-en-19-al, and 12-oxolabda-8(17), and 13E-dien-19 oic acid. Compared to the metabolites, among the acidic diterpene derivatives, five are considered majors, and were observed to show characteristic losses of 17/18, 43, and 59 Da, corresponding to the cleavage of HO^−^/H_2_O, CH_3_CO^−^, and CH_3_COO^−^ moieties, as evidenced in hydroxycopalic acid/(+)-isocupressic acid (peaks 17–20) [M−H]^−^ *m*/*z* at 319.2256, showing fragments at *m*/*z* 301.2172, 219.1386 and 217.1199, in 3keto-copalic acid (peaks 21–25) [M−H]^−^ *m*/*z* at 317.2095 at *m*/*z* 299.2042 and 273.1555, with a fragmentation pattern, and acetoxy copalic acid/acetylisocupressic acid (peaks 30-32) [M−H]^−^ *m*/*z* at 361.2385, showing mass fragments at *m*/*z* 301.2167, 219.1764, 217.8029, and 189.6338. 

One fatty acid (palmitic acid, peak 42) and a glucoside acid (Myristyl Glucoside, peak 41), representing the minor class detected in AHR extract, were eluted at the end of the chromatographic run (RT = 22.51 and 22.82 min) and are used as surface-active agents in sensitive skin cosmetics, whereas one flavonoid (peak 43) appeared in the last part of the chromatographic pattern (RT = 24.49 min). The low lipid content in AHR makes it an invaluable source to be used in the human diet. 

It is well known that the degree of liver injury correlates with the level of serum transaminases and ALP [55,56]. In the present study, administering a single dose of MTX induced hepatotoxicity, as evidenced by an elevation in serum ALT, AST, and ALP. This effect could be attributed to the generation of ROS and modulation of the activities of the antioxidant enzymes by MTX, with consequent oxidative stress and hepatocellular damage, as well as releasing liver enzyme markers in the blood [57]. In addition, MTX administration reduced the levels of SOD, HO-1, and NQO1, which are crucial components of the cellular defense mechanisms against oxidative stress and xenobiotic-induced toxicity [58]. Nrf2 is an important regulator of heme oxygenase-1 (HO-1) protein and antioxidative response element (ARE)-activated gene expression. Under physiological conditions, Nrf2 binds to Kelch-like ECH-associated protein-1 (Keap1). In oxidative stress, Nrf2 is released from Keap1 and quickly translocated to the nucleus, where it binds to ARE sequences, resulting in the transcriptional activation of antioxidant genes such as thioredoxin-1, HO-1, and NADPH quinone oxidoreductase-1 (NQO-1) [59]. On the other hand, pre-treatment using AHR markedly alleviated hepatocellular injury, as demonstrated by reduced levels of serum ALT, AST, and ALP. Correspondingly, AHR mitigated the disturbance in antioxidant defense mechanisms and oxidative stress, as manifested by increased levels of antioxidant enzymes SOD, HO-1, and NQO1, which are involved in the clearance of ROS, which could be attributed to their hepatoprotective effect. Nevertheless, AHR extract surpassed the standard drug silymarin in terms of antioxidant activity and attenuated the increase in serum hepatic markers.

TGF-β and NF-κB are two key mediators of inflammation in liver injury. TGF-β is a pleiotropic cytokine released by fibroblasts. It is known for its pro-fibrotic features, stimulating the production of extracellular matrix proteins and thereby promoting fibrosis [10]. In the current study, the administration of MTX demonstrated a marked elevation in the expression of TGF-β and NF-κB, which is in line with previous studies [60]. However, the inhibition of the TGF-β/NF-κB pathway was found to be crucial for the prevention of liver damage progression [61,62]. In the current study, treatment with AHR exhibited a protective effect against MILI, displayed by the reduced expression of TGF-β. Nevertheless, both dosages of AHR extract surpassed the standard drug silymarin in terms of decreasing TGF-β expression.

The NF-κB signaling pathway plays a central function in modulating the inflammatory and immune response [63]. Upon activation by different stimuli such as oxidative stress and pro-inflammatory cytokines, NF-κB translocates to the nucleus and induces the transcription of genes encoding for pro-inflammatory mediators such as TNF-α, IL-1β, and IL-6 [64]. In the current study, injection of MTX augmented the activation of NF-κB, consequently exacerbating liver inflammation by promoting inflammatory cytokines and chemokines including TNF-α, IL-1β, and IL-6. These results are in line with those of priorly published studies [64,65]. Oppositely, treatment with AHR mitigated the NF-κB signaling pathway, therefore diminishing inflammatory cell infiltration, including that by TNF-α, IL-1β, and IL-6, consequently inhibiting hepatic inflammation. The data from the current study are consistent with prior research findings, indicating the anti-inflammatory impact of AHR, which was mediated via inhibiting the transcription of pro-inflammatory cytokines through hindering NF-κB activation [19,23,66]. Nonetheless, the effect of both doses of AHR extract on the NF-κB signaling pathway surpassed the effect of the standard drug silymarin.

The JAK/STAT3 signaling pathway interacts with NF-kB and is crucial for cytokine signaling, contributing to hepatic damage and inflammation [15,67]. Increasing evidence suggests that IL-6 activates STAT3 via phosphorylation, which, in turn, translocates to the nucleus and enhances the transcription of downstream genes [68]. In addition, oxidative stress and JAK/STAT3 cross-regulation were earlier thought to be involved in MILI [5,69]. Recently, administration of MTX resulted in the activation of the JAK/STAT3 pathway, which was displayed by the increased gene expression of JAK and STAT3. Interestingly, treatment with AHR reduced JAK/STAT3 signaling, which might be attributed to the inhibition of IL-6 expression [23,70].

VEGF is a potent angiogenic factor that plays a critical role in promoting the formation of new blood vessels. A high level of VEGF is linked to an increased risk of liver disease and a poorer clinical outcome. The interaction of VEGF with its receptors induces angiogenesis, lymphangiogenesis, and increased vascular permeability [71]. Methotrexate-induced toxicity was previously reported to involve increased VEGF expression, thus promoting the extravasation of inflammatory cells and mediators into the tissue, thereby exacerbating tissue inflammation and injury [72]. Similarly, in the current study, injection of MTX displayed an elevation in VEGF expression. Nonetheless, a previous study reported that VEGF started to be overexpressed following 8 h of acetaminophen toxicity, indicating liver repair after drug-induced injury [73]. On the contrary, pre-treatment using AHR demonstrated a hepatoprotective effect, contributing to the decreased expression of VEGF compared to that in the diseased group. The hepatoprotective effect of AHR might be due to its polyphenols, which possess anti-proliferative and anti-angiogenesis properties against cancer cells in vitro [74,75]. 

Cyclin D1 is a key regulatory protein involved in controlling cell cycle progression from the G1 to the S phase, and its expression is regulated by STAT3 [76]. It was previously reported that cyclin D1 is overexpressed in the liver after DILI, which may be caused by the noxious effect of ROS and inflammation, resulting in cell stress and injury. The overexpression of cyclin D1 and VEGF highlights the liver’s attempt to repair and regenerate after injury [73]. Our findings demonstrated tissue stress and injury, which were revealed by the increased expression level of cyclin D1 after MTX administration. On the other hand, AHR demonstrated a downregulation in the expression of cyclin D1, explaining a part of its hepatoprotective effects. Previous studies on natural compounds suggested that a part of these compounds’ hepatoprotective impact could be attributed to the inhibition of cyclin D1.

P38, a member of the MAPK family, is known for its pivotal role in mediating cellular responses to diverse stressors, including oxidative stress and inflammatory cytokines [77]. Phosphorylation of p38 leads to its activation, allowing it to mediate a wide variety of cellular responses, including apoptosis and inflammation [13]. One of the mechanisms involved in the adverse effects of MTX encompasses the activation of p38 MAPK, followed by p38 MAPK-induced inhibition of Bcl-2, a pro-survival regulator of the apoptosis pathway in the mitochondria [18]. Our results confirm the findings from earlier studies showing that MTX-induced hepatic apoptosis is mediated through the p38 MAPK-induced downregulation in BCL2 [78,79]. On the other hand, AHR pre-treatment significantly decreased the expression of p38, accompanied with an elevation in BCL2 expression, indicating an anti-apoptotic mechanism as a part of the hepatoprotective effect of AHR. This effect might be related to the observed anti-inflammatory and antioxidant impact of AHR, since oxidative stress and inflammation are two mechanisms working in parallel in DILI to provoke liver cell apoptosis [66,80]. In this context, diterpenes are considered functional antibacterial, antiviral, anti-inflammatory, cytotoxic, and hepatoprotective agents, especially the labdane-type [81,82]. Overall, the extract’s impressive antioxidant and anti-inflammatory profile positions it as a compelling natural compound with promising implications for future research and clinical applications.

## 4. Materials and Methods

### 4.1. Chemicals 

Analytical-grade absolute ethanol was purchased from El-Gomhouria Company, Cairo-Egypt for the extraction method. In addition, for the chromatographic technique, acetonitrile, ammonium formate, formic acid, methanol, and sodium hydroxide for pH adjustment were purchased from Fisher Scientific, Loughborough, UK/Sigma-Aldrich, Hamburg, Germany. 

### 4.2. Plant Collection, Authentication, and Preparation of the Extract

AHR was collected during the flowering stage in April 2018 from El-Muntaza Palace Garden, Alexandria, Egypt. The plant was validated by a senior taxonomist in NRC (National Research Centre), Giza, Egypt, Dr. M. Gibali, and a senior botanist in Orman botanic garden, Giza, Egypt, Mrs. Therese Labib. It was deposited under voucher no Sp. # AH 2.7.2019. 

AHR (50 g) was scratched from the stem of the plant, dried at room temperature (27 °C ± 2), cut into small pieces, washed, grinded, and packed in a plastic bag. The fine powder was then soaked in 200 mL of absolute ethanol in a sonicator (Soltec Co., 230/240 V, 50/60 *Hz*, Milan, Italy) for 15 min. The filtrate was evaporated to dryness using a rotary evaporator (equipped with a water bath set to 50 ± 5 °C and a vacuum source). The crude extract was de-hydrated using anhydrous CaCl_2_ until a constant weight and then freeze-dried to provide a fine powder with a constant weight (31 g) to ensure the complete removal of any solvent traces or humidity and to ensure that the ethanol had no impact on hepatic injury in the experiment.

### 4.3. LC-HRMS/MS Analysis

The phytochemical profile for the AHR extract (50 mg) was determined based on Dalia et al. 2023 [66], with some updates. Acetonitrile: MeOH: H_2_O, 25: 25: 50, *v*/*v*/*v*, as a mobile phase was used to dissolve the resin. It was stirred for 2 min, and then for 10 min by vortex and ultrasonicate, respectively. The sample was centrifuged at 1000 rpm for 10 min until a final concentration of 2.5 µg/µL of the injected sample was achieved for both modes (negative and positive) to be applied on the Triple TOF 5600+ nano LC-HRMS/MS device. As documented in previous procedures in [28,29,66], HPLC and precolumn separations were achieved. Three solvents, A-C, for both or one of the two modes, were used during the whole analysis process. These included solvents A/B, 5 mM ammonium formate buffer, pH 3/8, containing 1% methanol for positive/negative modes, respectively, and solvent C, 100% acetonitrile for both modes, where at 0–1 min, isocratic elution (95–5% (A) or (B)–(C)) was observed, and from 1–28 min, a linear gradient from 95–5% to 5–95% of (A) or (B)–(C) at a rate of 0.3 mL/min at 40 °C was observed. From 28.1 to 35 min, elution was isocratic (95–5% (A) or (B)–(C)) (17,18). The mixture of solvents A and C was applied for the positive mode, while B and C solvents were applied for the negative mode. 

### 4.4. In Vivo Hepatoprotective Activity

#### 4.4.1. Animals

Male albino rats (150–170 gm, 2 months old) were obtained from the animal facility of Faculty of Pharmacy, Cairo University (Egypt). The animals were kept in an animal house at the Faculty of Pharmacy—Egyptian Russian University, at a room temperature of 25 ± 1 °C, with 12 h light and 12 h dark cycles. They had access to food and water for one week prior to the study for acclimatization. The experimental protocols were carried out in agreement with the guidelines accepted by the Egyptian Russian University Research Ethics Committee (REC-ERU), with approval number ERUFP-PO-24-002.

#### 4.4.2. Experimental Design

Rats were randomly separated into five groups (*n* = 6/group): Group I, which received 5% *v*/*v* Tween 80 in normal saline p.o. and were assigned as “normal control” group; Group II–V, where animals received a single dose of MTX (20 mg/Kg, i.p.; Sigma-Aldrich, MO, USA) on the 13th day [83]; Group III, which was treated with AHR (100 mg/kg/day; p.o.) [66]; Group IV, which was treated with AHR (200 mg/kg/day; p.o.) [66]; and Group V, which was treated with silymarin (100 mg/kg/day; p.o.; Sigma-Aldrich, St. Louis, MO, USA) [84]. All treatments were dissolved in 5% *v*/*v* Tween 80 in normal saline, administered via gastric gavage to ensure precise dosage delivery for a period of 2 weeks.

On the 15th day, anesthetization of rats was carried out using thiopental in a given dose (50 mg/kg, i.p.), and blood was obtained from the retro-orbital sinus. Separation of serum was achieved by centrifugating blood samples measuring 3000 g for 10 min then stored at −20 °C for further hepatic marker analysis. Meanwhile, the liver was isolated and split into two sections. One section was preserved in 10% formalin solution and used for histopathological examination and immunohistochemical assays, while the other part was used for further analysis using a colorimetric assay, an enzyme-linked immunosorbent assay (ELISA), and real-time quantitative reverse transcription polymerase chain reaction (RT-qPCR) assays.

#### 4.4.3. Evaluation of Hepatic Markers in Serum

Aspartate transaminase (AST) (cat#: 260001 Spectrum diagnostic, Cairo, Egypt), alanine transaminase (ALT) (cat#: 264001 Spectrum diagnostic, Cairo, Egypt) and alkaline phosphatase (ALP) (cat#: DALP-250 BioAssay systems, Hayward, CA, USA) levels were measured colorimetrically following the manufacturer’s guidelines. The results of AST, ALT, and ALP were presented as U/L.

#### 4.4.4. Evaluation of Oxidative Stress Markers in Liver Tissue

Colorimetric assay kits were used to assess super oxide dismutase (SOD) (Biodiagnostic, Cairo, Egypt), heme oxygenase 1 (HO-1) (Biovision, Milpitas, CA, USA), and NAD(P)H dehydrogenase [quinone] 1 (NQO1) (MyBioSource, San Diego, CA, USA) levels. The procedures were conducted in accordance with the manufacturer’s guidelines, and the results ae presented as U/mg tissue protein for SOD, as ng/mg tissue protein for HO-1, and as pg/mg tissue protein for NQO1.

#### 4.4.5. Evaluation of NF-κB, IL-1β, IL-6, TNF-α and Cyclin D in Liver Tissue

ELISA kits (Cloud-Clone Corp., Katy, TX, USA) were utilized to assess NF-κB (cat#: SEB824Ra), IL-1β (cat#: SEA563Ra), IL-6 (cat#: SEA079Ra), TNF-α (cat#: SEA133Ra), and Cyclin D (cat#: SEA585Ra). The methods were conducted according to the manufacturer’s protocol; the results are expressed as ng/mg tissue protein for NF-κB and Cyclin D, and as pg/mg tissue protein for IL-1β, IL-6, and TNF-α.

#### 4.4.6. Evaluation of JAK, STAT3, p38, and BCL2 in Liver Tissue

For the assessment of the mRNA expression of JAK, STAT3, p38, and BCL2, liver specimens were homogenized in lysate buffer. For the isolation of total RNA, the RN easy mini kit was utilized, and purity was assessed using a spectrophotometer at wavelength of 260–280 nm. Transcription of cDNA was carried out according to the manufacturer’s protocol (Promega, Leiden, The Netherlands). Quantification using RT-PCR was executed for JAK, STAT3, p38, and BCL2 according to the instructions of SYBR Green Master Mix (Applied Biosystems, Foster City, CA, USA). The primer sequences are displayed in Table 2. Thermal cycling was carried out in 40 cycles for the completion of PCR amplifications, at 95 °C for 15 s, at 60 °C for 60 s, and at 72 °C for 60 s. Following the completion of the RT-PCR run, the results were quantified using the cycle threshold (Ct) method. The results are displayed as relative fold changes in comparison to expression of control gene (GAPDH).

#### 4.4.7. Histopathological Examination

Liver samples were collected and preserved in a 10% solution of neutral buffer formalin for twenty-four hours. The washing process involved using tap water, followed by the use of ethyl alcohol in serial dilutions for dehydration. The specimens underwent xylene clearance and paraffin embedding. Paraffin-embedded tissue blocks were prepared for sectioning at a thickness of 4 microns using a sliding microtome. The tissue sections were stained with hematoxylin and eosin stain for evaluation [85].

#### 4.4.8. Immunohistochemical Detection of TGF-β and VEGF

The paraffin sections were affixed to slides with a positive charge using the avidin biotin–peroxidase complex (ABC) technique. Sections from each group were subjected to incubation with the primary ntibodies TGF-β (cat#: A15103 ABclonal, Woburn, MA, USA) and VEGF (cat#: A0280 ABclonal, MA, USA) at a 1:100 dilution, followed by the addition of the necessary reagents for the ABC method using the Vectastain ABC-HRP kit, Vector labs. The marker expression was labeled using peroxidase and stained with diaminobenzidine (DAB, Sigma-Aldrich, St. Louis, MO, USA), in order to detect the antigen–antibody complex. Non-immune serum was used as a negative control instead of the primary or secondary antibodies. The IHC stained sections were viewed using an Olympus microscope (model BX-53). Scoring of immunohistochemistry results was carried out by determining the reaction area percent in 10 microscopic fields using software version 1.53t (ImageJ, NIH, USA).

#### 4.4.9. Statistical Analysis

Data are displayed as the mean ± SD (number per group = 6 rats) using one way ANOVA followed by Tukey’s post hoc test, at a *p* value < 0.05.

## 5. Conclusions

In conclusion, this study is the first to examine the hepatoprotective effects of AHR in MILI based on previous reports mentioning its anti-inflammatory and antioxidant effects. Pre-treatment with AHR showed hepatoprotective effects against MILI through anti-inflammatory, antioxidant, anti-angiogenic, and anti-apoptotic mechanisms. These mechanisms are mediated through the inhibition of the TGF-β/NF-κB/TNF-α/IL-1β pathway, as well as IL-6/JAK/STAT-3/cyclin D1/VEGF, and the modulation of the p38 MAPK/BCL2 cascade. Also in the present study, LC-HR-MS/MS was utilized for the analysis of the Egyptian cultivar AHR. Collectively, 40 metabolites were characterized in ethanolic extract, with two fatty acid compounds and one flavonoid;17 metabolites were unreported in the *Araucaria* genus. The observed compounds were classified into acidic-, phenolic-, volatile- diterpenes and others. All the characterized diterpenes were of the labdane, abietane, and clerodane, types, as in the *Araucaria species*. Moreover, this is the first report observing 15-formyloxylabd-8(17)-en-19-oic acid as a natural metabolite in the AHR extract. The promising antioxidant and anti-inflammatory activities observed in this study suggest that this extract could be a valuable adjunct in the management of liver injury, particularly when used in combination with conventional treatments. Accordingly, the alcoholic extract of AHR could be a promising alternative with which to produce hepatoprotective agents; support via further studies is required to detect the most important bioactive metabolites responsible for the in vivo hepatoprotective effects observed, including pharmacodynamic and pharmacokinetic studies.

## Figures and Tables

**Table 2:** Primers used for RT-qPCR.

mRNA Species	Accession Number	Sequence (5′→3′)
JAK	ON706994	F: TTTGGATCCCTGGATACATACCTGA R: TGGCACACACATTCCCATGA
STAT3	XM_054316993	F: CCCCGTACCTGAAGACCAAGT R: CCGTTATTTCCAAACTGCATCA
P38	NM_001109891	F: TCATAGGCATCCGAGACATCC R: CGTCTCCATGAGGTCCTGAAC
BCL2	XM_047437733	F: ATCGCTCTGTGGATGACTGAGTAC R: AGAGACAGCCAGGAGAAATCAAAC
GAPDH	NM_002046.7	F: GTCTCCTCTGACTTCAACAGCG R: ACCACCCTGTTGCTGTAGCCAA

JAK, Janus kinase; STAT3, signal transducers and activators of transcription 3; BCL2, B-cell lymphoma 2; GAPDH, glyceraldehyde 3-phosphate dehydrogenase.

## Data Availability

Data are contained within the article.
